# The effects of changes in flowering plant composition caused by nitrogen and phosphorus enrichment on plant–pollinator interactions in a Tibetan alpine grassland

**DOI:** 10.3389/fpls.2022.964109

**Published:** 2022-07-25

**Authors:** Lin-Lin Wang, Fei Ren, Chan Zhang, Xiao-Juan Huang, Zhen-Hua Zhang, Jin-Sheng He, Yong-Ping Yang, Yuan-Wen Duan

**Affiliations:** ^1^Germplasm Bank of Wild Species, Kunming Institute of Botany, Chinese Academy of Sciences, Kunming, China; ^2^Institute of Tibetan Plateau Research at Kunming, Kunming Institute of Botany, Chinese Academy of Sciences, Kunming, China; ^3^State Key Laboratory of Plateau Ecology and Agriculture, Qinghai University, Xining, China; ^4^College of Life Sciences, Henan Normal University, Xinxiang, China; ^5^College of Life Sciences, Northwest University, Xi’an, China; ^6^University of Chinese Academy of Sciences, Beijing, China; ^7^Key Laboratory of Adaptation and Evolution of Plateau Biota, Haibei Alpine Grassland Ecosystem Research Station, Northwest Institute of Plateau Biology, Chinese Academy of Sciences, Xining, China; ^8^Department of Ecology, Key Laboratory for Earth Surface Processes of the Ministry of Education, Peking University, Beijing, China; ^9^State Key Laboratory of Grassland Agro-Ecosystems, College of Pastoral Agriculture Science and Technology, Lanzhou University, Lanzhou, China

**Keywords:** climate change, grassland ecosystems, biodiversity loss, nutrition addition, pollination network, Qinghai-Tibet Plateau

## Abstract

Soil eutrophication from atmospheric deposition and fertilization threatens biodiversity and the functioning of terrestrial ecosystems worldwide. Increases in soil nitrogen (N) and phosphorus (P) content can alter the biomass and structure of plant communities in grassland ecosystems; however, the impact of these changes on plant–pollinator interactions is not yet clear. In this study, we tested how changes in flowering plant diversity and composition due to N and P enrichment affected pollinator communities and pollination interactions. Our experiments, conducted in a Tibetan alpine grassland, included four fertilization treatments: N (10 g N m^–2^ year^–1^), P (5 g P m^–2^ year^–1^), a combination of N and P (N + P), and control. We found that changes in flowering plant composition and diversity under the N and P treatments did not alter the pollinator richness or abundance. The N and P treatments also had limited effects on the plant–pollinator interactions, including the interaction numbers, visit numbers, plant and pollinator species dissimilarity, plant–pollinator interaction dissimilarity, average number of pollinator species attracted by each plant species (vulnerability), and average number of plant species visited by each pollinator species (generality). However, the N + P treatment increased the species and interaction dissimilarity in flowering plant and pollinator communities and decreased the generality in plant–pollinator interactions. These data highlight that changes in flowering plants caused by N + P enrichment alter pollination interactions between flowering plants and pollinators. Owing to changes in flowering plant communities, the plant–pollinator interactions could be sensitive to the changing environment in alpine regions.

## Introduction

The structure and function of biodiversity, such as the biomass and diversity of plants and the animals that depend on them for survival, are vital for the sustainability of ecosystems (McCann, 2007). However, biodiversity loss caused by anthropogenic nutrient enrichment and climate change threatens the functions and services of terrestrial ecosystems, particularly grasslands (Hautier et al., 2009; Hooper et al., 2012; Isbell et al., 2013). For example, land-use change and environmental pollution have contributed to a decline in biodiversity worldwide (IPBES, 2018). To restore grassland services and functions, specific agricultural management strategies, such as livestock exclusion and chemical fertilizer application, are employed to improve grassland productivity and other ecological services, such as pollination (IPBES, 2016). Plant–pollinator interactions can change through an increase in dominance and a decrease in the diversity of plants in grasslands under nutrient enrichment through agricultural fertilization and atmospheric deposition (Zarzycki and Kopec, 2020; Villa-Galaviz et al., 2021). In addition, the number and diversity of wild pollinators in natural and agricultural ecosystems have declined because of climate change, habitat loss, pathogen transport, commercially managed pollinators, agrochemicals, and nutrient enrichment (Potts et al., 2010; Ghazoul, 2015; Baude et al., 2016; Ollerton, 2017), which alter the stability of pollination ecosystems. However, since recent reviews predict that N enrichment might disrupt or enhance individual plant–pollinator interactions in grassland communities (Stevens et al., 2018; David et al., 2019), it is not yet clear how the changes in plants due to multiple nutrient enrichment in soil alter plant–pollinator interaction networks.

The availability of nitrogen (N) is considered to be the most important nutritionally limiting factor for primary productivity in grasslands (Elser et al., 2007; Harpole et al., 2011; Xiao et al., 2020). Although the N supply is increasing worldwide (Galloway et al., 2008; Liu et al., 2013; Wang et al., 2017), N availability has decreased in many regions of the world due to increased temperatures and CO_2_ (Mason et al., 2022; Olff et al., 2022), dramatically reducing the biodiversity of terrestrial ecosystems (Hooper et al., 2012; Storkey et al., 2015). Nutrient co-limitation is common in the biomass of plant communities (Elser et al., 2007; Harpole et al., 2011). Phosphorus (P) enrichment can increase soil P bioavailability and accelerate N uptake by plants, which can increase plant biomass and change the structure of plant communities (Ren et al., 2017; Xiao et al., 2020). This reshaping of the composition of plant communities through nutrient enrichment can change the animal and microbial diversity and abundance above and below ground (Liu et al., 2021; Villa-Galaviz et al., 2021; Zi et al., 2022), in addition to potentially altering biodiversity at multiple trophic levels within the community’s food webs (Tylianakis et al., 2008; Burkle and Irwin, 2009; Xiao et al., 2020; Villa-Galaviz et al., 2021). Some studies on the responses of pollinator communities and plant–pollinator interaction networks to nutrient supply in grassland ecosystems have been conducted, but their results have been inconsistent (Burkle and Irwin, 2009; Carvalheiro et al., 2019; Villa-Galaviz et al., 2021). For example, Burkle and Irwin (2009) found that N enrichment exerted no effect on pollinator assemblages and the community network structure between plants and pollinators, but Villa-Galaviz et al. (2021) showed that fertilizer addition decreased plant species richness, floral abundance and bumblebee richness. Therefore, there is an urgent need to focus on the consequences of soil nutrient intake on pollinator assemblages and plant–pollinator interaction networks in other grassland ecosystems from agricultural activities and environmental changes.

The Tibetan grassland ecosystem covers over 60% of the Qinghai-Tibet Plateau; however, this region is experiencing increased atmospheric N deposition and local warming (Chen et al., 2013; Liu et al., 2013). These grasslands are fragile ecosystems owing to their high altitude and low temperature and are sensitive to human activities and global changes (He et al., 2016; Liu et al., 2021; Dong et al., 2022). Previous studies have shown that adding N and P can increase the net primary productivity of grasses, while predominantly reducing the biomass and abundance of legumes on the alpine grasslands (Ren et al., 2016; Ren et al., 2017; Luo et al., 2019). Many recent studies have focused on the effects of N and P addition on plants and soil microorganisms in the alpine grasslands (Zong and Shi, 2019; Liu et al., 2021; Dong et al., 2022; Zi et al., 2022), showing that combined N and P enrichment decreased the diversity of plants but increased net primary productivity (Ceulemans et al., 2013; Xiao et al., 2020). However, because bottom-up effects driven by changes in flowering plant diversity and flower abundance could have a major impact on the plant–pollinator interactions, few studies have focused on how changes in plant community structure due to N and P enrichment affect the richness and abundance of pollinators in this alpine grassland ecosystem.

**FIGURE 1:**
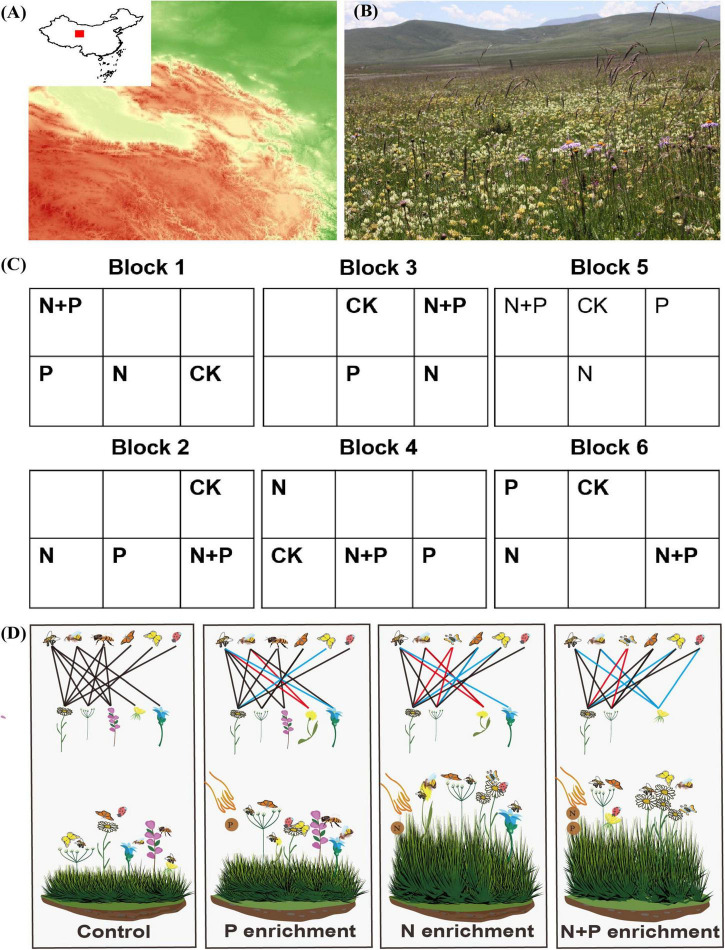
Description of study area and experiment design. **(A)** Location of the study area in the northeastern Qinghai-Tibet Plateau, Qinghai, China. **(B)** Alpine grassland landscape of the study site and legumes, which are in full bloom (taken by Lin-Lin Wang, July 30, 2017). **(C)** Study design shows 24 plots in the N and P enrichment plots. CK represents the control plots. Two empty plots in each block indicate not included in this experiment. **(D)** Schematic framework of how N and P enrichment affects the diversity of flowering plants, pollinator communities, and plant–pollinator interactions. The colored links identify the changes in plant and pollinator interactions due to nutrient enrichment. Pictures of **(D)** are available at https://pixabay.com.

Here, we experimentally manipulated the addition of nutrients to investigate whether and how the addition of P and N affected flowering plant communities, pollinator assemblages, and plant–pollinator interactions (Figure 1). In addition, we used a network approach to describe the interaction networks between plants and pollinators, which is commonly used to study changes in plant–pollinator interactions in communities (Kaiser-Bunbury et al., 2017; Gao et al., 2021; Villa-Galaviz et al., 2021). Previous research showed that nutrient addition reduced the diversity of plants in grasslands (Stevens et al., 2018; Xiao et al., 2020; Villa-Galaviz et al., 2021). Therefore, we hypothesized (Figure 1D) that the co-addition of N and P (1) would decrease the diversity of flowering plants and alter the composition of flowering plant communities (Hypothesis 1); (2) decrease the diversity of pollinators and change the structure of pollinator assemblages due to reduced legume biomass and relative abundance (Hypothesis 2); and (3) change plant–pollinator interactions across trophic levels due to changes in the relative abundances of flowering plants and pollinators (Hypothesis 3). The insights gained from this research will improve our understanding of how global changes, such as atmospheric nitrogen deposition and fertilization, can maintain and improve ecosystem biodiversity and the structure of interaction networks between plants and pollinators and make grassland ecosystems more sustainable.

## Materials and methods

### Study area

This study was conducted at the Haibei Alpine Grassland Ecosystem Research Station (37° 36′ N, 101° 12′ E, 3,250 m above sea level), Qinghai, China (Figure 1A). The research station has a typical plateau continental climate (short and cool summers, and long and cold winters), with a mean annual temperature and precipitation of –1.1°C and 480 mm, respectively. The precipitation (about 80%) is mainly concentrated in the growing season (May–September). The soil in this area is classified as Mat-Gryic Cambisols (WRB, 1998). In 2011, the soil content 10 cm underground in the station sampling site contained total carbon, 78.2 g kg^–1^; organic carbon, 63.1 g kg^–1^; nitrogen, 5.75 g kg^–1^; and phosphorus, 0.79 g kg^–1^ (Zhang et al., 2021). The plant community mainly consists of grasses and forbs (Figure 1B). The flowering plant species are mainly *Gentiana straminea*, *Gentiana aristata*, *Morina kokonorica*, *Angelica nitida*, and *Dasiphora fruticosa* (Liu et al., 2019), which are primarily pollinated by insects such as honey bees, bumble bees, butterflies and flies (Zhang et al., 2015; Wang et al., 2021).

### Nitrogen and phosphorus manipulations

From May 2011, four nutrient-addition experiments were conducted in an alpine grassland: N addition, P addition, a combination of N and P addition, and no nutrient addition (Figure 1C). The fertilizers were 100 kg of urea (CH_4_N_2_O) and 50 kg of triple superphosphate [Ca(H_2_PO_4_)_2_⋅CaHPO_4_] per hectare per year. Our experiment included 24 plots (4 treatments × 6 blocks) at 6 m × 6 m each (Figure 1C), which is comparable to other studies (Burkle and Irwin, 2009; Villa-Galaviz et al., 2021). In the experimental plots, the legumes and forbs mainly include *Oxytropis kansuensis*, *Gentiana straminea, A. nitida, Aster farreri*, *Tibetia himalaica*, and *Thermopsis lanceolata*. To maintain the plant species’ similarity and reduce the impairment caused by different fertilization treatments on adjacent plots, the blocks were spaced 2 m apart, and the plots within each block were spaced 1 m apart. We divided the fertilizer into three portions and spread it evenly from June to August each year (Ren et al., 2017).

### Flower and pollinator survey

To examine the change in the diversity and flower abundance of flowering plants after the N and P addition treatments for eight consecutive years (2011–2018), we created four transects (1 m × 5 m) within each plot in 2019. From early July to late August 2019, we counted the number of flowering plant species and flowers for each plant four times per half month along the four transects of each plot.

To examine the diversity and number of pollinating insects, from early July to late August 2019, we monitored flowers for 30 min in each transect (1 m × 5 m) four times on clear days in the presence of strong wind (09: 00–19: 00). Using this observation method, each plant species was observed in each transect for 120 min. If an insect encountered the sexual components of the flowers (anthers or stigmas), we recorded a pollination visit without considering the effectiveness of the pollination (Memmott, 1999). As few pollinators visited the flowers in alpine meadows at low temperatures, we did not observe pollinator visits at night (Fang and Huang, 2016). The pollinator abundance depended on the number of visits to flowering plants per plot for each pollinator species. With the help of taxonomic experts, we identified the species or higher-level groups of unknown visitors.

### Pollination network construction and parameter calculation

To estimate the completeness of the sample, we divided the observed richness by the estimated abundance-based richness estimator, Chao1, using the vegan package (v2.5-7) with the R statistical software (v4.0.3, R Core Team, 2020).

To determine the effects of nutrient addition on the diversity and abundance of flowering plants and pollinators, we counted the flowering plant species, pollinator species, flowers, pollinator individuals, plant–pollinator unique interactions, and visits per plot.

To determine whether the core generalist flowering plant and pollinator species changed with different nutrient treatments, we used UCINET (v6.0) to calculate the eigenvector centrality score (network > centrality > eigenvector in UCINET) of each plant and pollinator species of different nutrient treatments (Alarcón et al., 2008). In addition, when one plant or pollinator species with a high eigenvector centrality score participated in more than 5% of the visits and interacted with more than 25% of the taxa in each nutrient treatment, we delimited them as core generalists (Alarcón et al., 2008). We then analyzed the core generalized plant and pollinator species to determine whether they had changed due to the nutrient addition treatments.

To determine the effects of nutrient addition on the dissimilarity between flowering plants and pollinators, we quantified the species and interaction dissimilarity (species or link differences in plant–pollinator interaction networks) based on the methods of Poisot et al. (2012). We ran all combinations of networks using the “betalinkr” function in the bipartite package (Dormann et al., 2021). According to Poisot et al. (2012), the dissimilarity of a pair of networks was divided into the dissimilarity due to differences in species composition (species dissimilarity) and dissimilarity due to interaction rewiring (interaction dissimilarity). We focused on the change in species and interaction composition involving species of plants and pollinators that were present for the control and nutrient-addition treatments. These two metrics quantify species and link changes in species interaction networks under different nutritional treatments (Poisot et al., 2012). A value of 0 indicates that the species/interaction compositions are identical, and a value of 1 indicates that the species/interaction compositions are completely different.

To determine the impact of nutrient addition on the changes in links between plants and pollinators, we calculated two quantitative network metrics, vulnerability and generality, using the methods of Villa-Galaviz et al. (2021). We used the “networklevel” function in the bipartite package (Dormann et al., 2021) to calculate the values of vulnerability and generality. The vulnerability and generality were weighted according to their marginal sums (Bersier et al., 2002). The vulnerability corresponds to the average number of pollinator species attracted by each plant species, and the generality corresponds to the average number of plant species visited by each pollinator species (Villa-Galaviz et al., 2021). The changes in vulnerability and generality reflect the effects of nutrient addition on the distribution of links between plants and pollinators (Villa-Galaviz et al., 2021). An increase in vulnerability indicates that pollinator communities are increasingly dependent on fewer plant species. Conversely, an increase in generality demonstrates that the range of plant species visited by pollinators has increased, or that generalized pollinator species have increased their abundance.

### Data analysis

The data were checked for normality and homogeneity of variance before performing the ANOVA. If the assumptions of normality and variance were not met, the data were log-transformed. We then used two-way ANOVAs in the R basic package to determine the effects of nutrient enrichment on plants, pollinators, and their interactions. The N and P treatments were fixed factors, and the block was an error term. The variables included five flowering plant and pollinator variables (the flowering plant diversity, flower abundance, pollinator diversity, pollinator abundance, and species dissimilarity of plants and pollinators) and five pollination interaction variables (the interaction numbers, number of visits, interaction dissimilarity, vulnerability, and generality). In addition, three other statistical analyses (one-way ANOVA, the pairwise permutational multivariate analysis of variance, and piecewise structural equation modeling) were performed.

First, we used a one-way ANOVA to examine the effects of the nutrient-addition treatments (control, N, P, and N + P treatments) on each response variable (the flowering plant diversity, pollinator diversity, flower abundance, pollinator abundance, interaction numbers, number of visits, species dissimilarity, interaction dissimilarity, vulnerability, and generality).

Second, we applied the pairwise permutational multivariate analysis of variance (PERMANOVA) using the vegan package (v2.5-7) (Oksanen et al., 2020) to evaluate the effects of the addition of N and P on the plant and pollinator diversity. To illustrate the impacts of the N and P treatments on changes in the structure of plant and pollinator communities, we performed paired Bray–Curtis distance principal coordinate analysis (PCoA).

Finally, we applied piecewise structural equation modeling in the piecewise SEM package (v2.1.2) (Lefcheck, 2016) to clarify the direct and indirect effects of the addition of N and P on changes in plant–pollinator interactions (Supplementary Figure 1). The hypothesized direct effects of N and P enrichment, and the indirect effects mediated by plant and pollinator community (i.e., plant diversity, flower abundance, pollinator diversity, and pollinator abundance) on vulnerability and generality are included in Supplementary Figure 1. The piecewise SEM included several linear mixed-effects models, and the block was a random effect. The full piecewise SEM comprised the effects of N and P’s effects on four mediators of plant and pollinator communities (plant diversity, flower abundance, pollinator diversity, and pollinator abundance), as well as the direct and indirect effects on the vulnerability and generality of plants and pollinators. To simplify the model, we did not consider the interactions between the N and P treatments.

## Results

**FIGURE 2:**
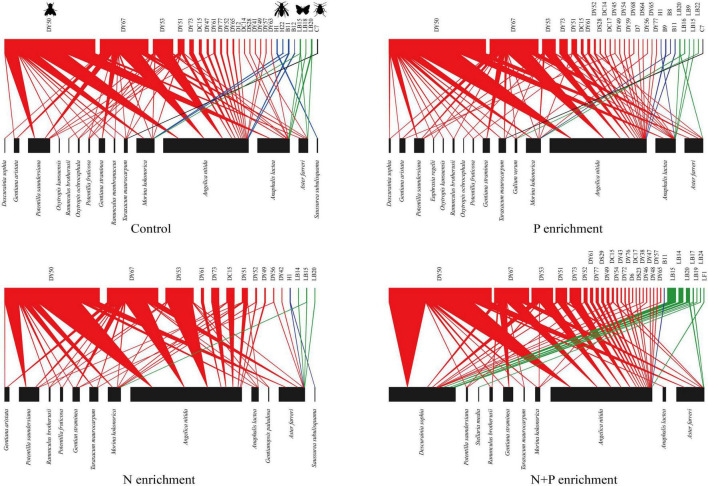
Plant–pollinator interaction networks for N and P enrichment on an alpine grassland. These pollination networks include flowering plant species (bottom blocks), pollinator species (top blocks), and their pollination interactions (color triangles). The widths of the blocks and triangles reflect the numbers of plants and pollinators or the numbers of pollination interactions, respectively. Colors represent the pollinator groups: green, Hymenoptera; red, Diptera; blue, Lepidoptera; black, Coleoptera.

In total, we found that 20 flowering plant species (Supplementary Table 1) were visited by 54 pollinator species (Supplementary Table 2) that participated in 4954 individual interactions (Figure 2). Based on the sample completeness analysis, these data represented about 64% of the pollinator species for each treatment.

### Structural changes from N and P addition to flowering plant communities

The N treatment decreased the flowering plant diversity, and the N + P treatment increased the negative effect on the flowering plant diversity (Figure 2 and Supplementary Figure 2A). In particular, the N treatment reduced the legume diversity (Figure 2, e.g., *O. kansuensis*, *Oxytropis ochrocephala*, and *Oxytropis qinghaiensis*). By contrast, the N + P treatment further reduced the diversity of rare species (Figure 2, e.g., *Ranunculus membranaceus*, *Euphrasia regelii*, and *Galium verum*). The abundance of flowers was similar to that for the control and N or P treatments (Supplementary Figure 2B). However, the N + P treatment increased the abundance of flowers (Supplementary Figure 2B), primarily by increasing the number of flowers of *Descurainia sophia*.

**FIGURE 3:**
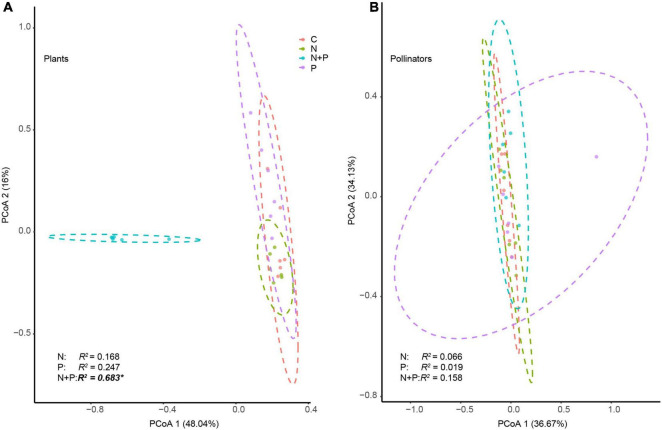
The effects of nutrient enrichment on the community structure of plant **(A)** and pollinator species **(B)**. The structure is based on a principal coordinate analysis. Permutation analysis of variance was used to evaluate the influence of N addition, P addition, and N and P co-addition on the community structure of plants and pollinators. The asterisk (*) indicates a significant difference at the 0.05 level.

The N + P treatment altered the numbers and identities of the core generalist plants (Supplementary Figure 3A). For example, four plant species (*Morina kokonorica*, *A. nitida*, *Anaphalis lacteal*, and *Aster farreri*) were core generalists with the control treatment. By contrast, only two species (*A. nitida* and *A. lacteal*) were core generalists with the P treatment (Supplementary Figure 3A). Additionally, *Potentilla saundersiana* dominated the N treatment group as the core plant species at the expense of *A. nitida*. To the detriment of *M. kokonorica* and *A. lacteal*, *D. sophia* became a core plant species in the N + P treatment (Supplementary Figure 3A). PERMANOVA analyses revealed that the N + P treatment changed the structure of the flowering plant communities (Figure 3A).

### Effects of structural changes in flowering plants on pollinator communities

Flies were the most abundant pollinators (Figure 2). The N and P treatments did not affect the diversity or abundance of pollinators (Supplementary Figures 2C,D). The N, P, and N + P treatments did not change the numbers and identities of the core pollinators (Supplementary Figure 3B), except that the N treatment decreased the diversity of the bees and the N + P treatment increased the diversity of the butterflies (Figure 2). None of the nutrient additions changed the structure of the pollinator communities (Figure 3B).

### The impacts of nutrient additions on plant–pollinator interactions

**FIGURE 4:**
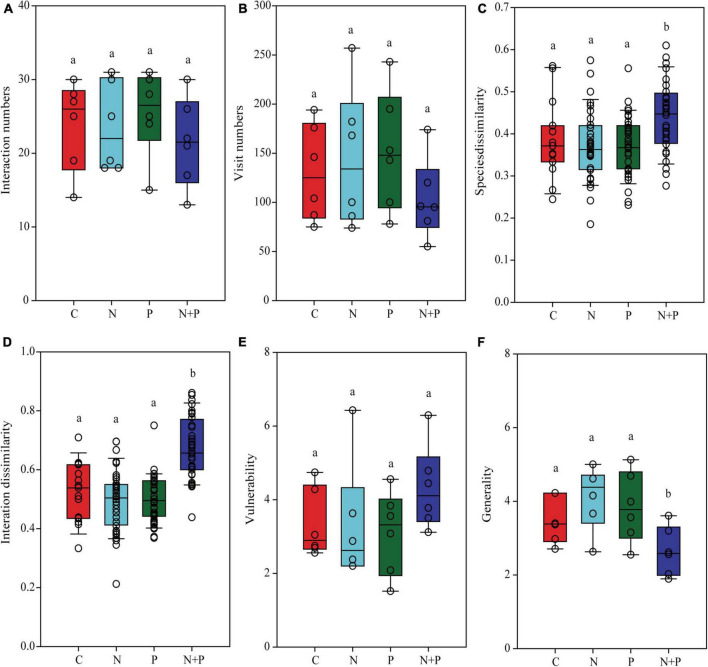
The effects of nutrient enrichment on the links between plants and pollinators. **(A)** plant–pollinator interaction numbers, **(B)** pollinator visit numbers, **(C)** plant–pollinator species dissimilarity, **(D)** plant–pollinator interaction dissimilarity, **(E)** vulnerability, and **(F)** generality. Each panel shows a one-way analysis of variance used to examine the differences between different nutrient treatments. Different lowercase letters on the bars indicate significant differences in nutrient supply at the 0.05 level.

The N, P, and N + P treatments did not change the number of unique interactions between plants and pollinators (Figure 4A) or the total number of visits to the pollination networks (Figure 4B). The N or P treatment alone did not change the species and interaction dissimilarity. However, compared to the control treatment, the N + P treatment increased the species and interaction dissimilarity in the pollination networks (Figures 4C,D). None of the treatments altered the vulnerability (Figure 4E). However, the N + P treatment decreased the generality (Figure 4F).

**FIGURE 5:**
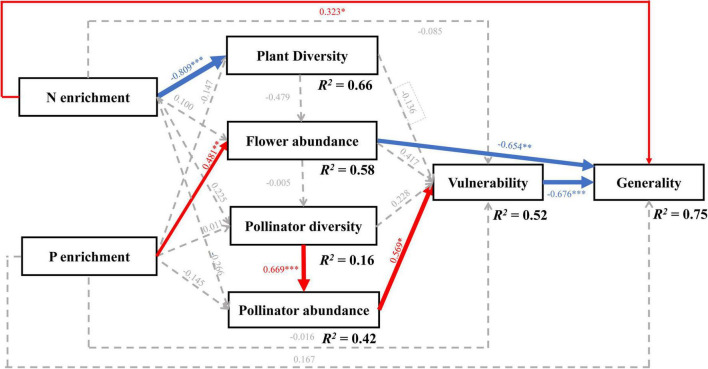
The results of the final piecewise structural equation model (SEM). The final SEM fitted the data: χ^2^ = 7.819, df = 14, *P* = 0.899, and AICc = –123.88. The numbers on the arrows indicate standardized path coefficients, and the asterisks show statistical significance: ^***^P < 0.001; ^**^P < 0.01; *P < 0.05. Red, blue, and gray arrows indicate positive, negative, and insignificant relationships. The arrow width shows the strength of the relationship, and the marginal *R*^2^ shows the variance, which is explained by fixed effects in the model.

The full piecewise SEM (Supplementary Figure 4 and Supplementary Table 3; χ^2^ = 1.629, df = 6, *P* = 0.95, and AICc = –108.163) and the final model are qualitatively similar (Figure 5 and Supplementary Table 3; χ2 = 7.819, df = 14, *P* = 0.899, AICc = –123.88, and ΔAICc = 15.72), which explained about 75% of the variance of the generality (marginal *R*^2^ = 0.75). The final model revealed that the change in flowering plant species caused by the N and P treatments indirectly affected the generality through changing the flower abundance and vulnerability (Figure 5 and Supplementary Figure 4). The N or P treatment alone did not affect the richness and abundance of the pollinators and vulnerability (all *P* > 0.05, Figure 5). However, the N treatment directly decreased the flowering plant diversity (standardized path coefficient β = –0.809, *P* < 0.0001) and increased the generality (β = 0.323, *P* = 0.03). In addition, the P treatment directly increased the abundance of flowers (β = 0.481, *P* = 0.004) and deceased the generality (β = –0.654, *P* = 0.001).

## Discussion

Examining how eutrophication due to climate change and anthropogenic activities affects communities of plants, animals, and species interactions is vital for understanding how biological communities respond to global changes (David et al., 2019). In the current study, we investigated the effects of N and P addition on plant–pollinator interactions in an alpine grassland. Our findings revealed that N and P addition changed the structure of flowering plant communities. Although pollinator communities were resilient to nutrient addition, the plant–pollinator interactions were unstable under the N + P treatment in alpine grassland ecosystems. These results suggest that N- and P-fertilization-induced changes in flowering plant composition have a minor effect on pollinator communities. However, the plant–pollinator interactions can be sensitive to nutrient addition through agricultural activities and environmental changes.

### Structural changes in flowering plant communities under N and P addition

Soil eutrophication due to N and P addition can increase the biomass of grasses and decrease the biomass and diversity of flowering plant species, such as forb and legumes, in nutrient-limited grasslands (Harpole et al., 2016; David et al., 2019). For example, fast-growing grasses can become taller, denser, and more competitive for sunlight under N enrichment. As a result, the biomass and diversity of nitrophobous forb species are often reduced and they can become locally extinct because of their small sizes and slow growth rates (Crawley et al., 2005; Storkey et al., 2015). The results of the present study support our first hypothesis that N enrichment reduces the diversity of flowering plants, primarily that of legumes (Figure 2). However, our findings did not agree with those of Ren et al. (2017), who found that the richness of legumes did not change under N enrichment in alpine grassland (Ren et al., 2017). A possible explanation is that the diversity of legumes deceased and disappeared over time, suggesting that the time and intensity of N addition played a vital role in their local extinction. For example, the effect of fertilization was not fully visible within only three years (Ren et al., 2017) because the perennial legumes (e.g., *O. kansuensis*, *O. ochrocephala*, and *O. qinghaiensis*) can only change their distribution over generations, so they can become locally extinct under long-term fertilization.

Additionally, this research revealed that P addition further enhanced the negative effect of N enrichment on flowering plant species in alpine grasslands (Figure 2). For example, *R. membranaceus*, *E. regelii*, and *G. verum* disappeared after eight years of N + P enrichment. N enrichment affects the N and P balance in the soil, changing the situation from N limitation to P limitation (Hooper et al., 2012; Harpole et al., 2016; Zhan et al., 2017). As P addition can also reduce the limitations for crucial nutrient resources (David et al., 2019), this could consequently alter the competitive dynamics of plants, typically leading to a reduction in legumes and endangered forbs (Suding et al., 2005; Clark et al., 2007). For example, N and P co-addition increased flower production because of the increased number of *D. sophia* plants (Supplementary Figure 2B), as N enrichment can significantly increase the height, biomass, and seed yield of *D. sophia* (Mokhtassi-Bidgoli et al., 2013). Furthermore, the changes in flowering plant diversity and flower abundance led to a further reorganization of the flowering plant community structure (Figure 3A), indicating that flowering plant communities are unstable under long-term P and N enrichment in alpine grassland ecosystems. However, future experiments with more time scales and fertilization intensities are still needed to examine the effects of nutrient enrichment on the structure of plant communities.

### Weak effects of structural changes in flowering plants on pollinator communities

Contrary to our second hypothesis, our findings revealed that N and P addition did not alter the pollinator diversity and abundance and the core pollinator species (Supplementary Figures 2, 3). Burkle and Irwin (2009) also found that N addition did not affect the richness of pollinator communities. A possible explanation might be that most of the remaining flowering plant species, such as Asteraceae, Ranunculaceae, and Rosaceae, had an open morphology with radially symmetrical flowers in the nutrient-addition plots (Figure 2 and Supplementary Table 1), which could attract many generalized pollinator species to visit these flowering plants (Duan et al., 2007; Zhang et al., 2015; Wang et al., 2021). For example, we found that the flowers of *D. sophia* and *Angelica nitida* could attract more than ten pollinator species. Additionally, our results showed that the main pollinator species were flies, bees, and butterflies (Figure 2), which are generalized pollinators that can visit many flowering plants in alpine grasslands (Wang et al., 2021). Therefore, we could not determine any changes in pollinator diversity and abundance due to P and N addition (Burkle and Irwin, 2009; Villa-Galaviz et al., 2021).

It is important to note that, similarly to in other studies (Supplementary Table 5), the scale of the experimental treatments for the plants was relatively small, which would probably have not affected the populations of pollinators but, rather, simply their behavior in terms of which plots they decided to forage in. However, our results showed that N and P co-addition decreased the richness of bumble bees but increased the diversity of butterflies (Figure 2). Changes in the flowering plant species composition can negate the ability of pollinators to choose different resource supplies (Burkle and Irwin, 2009; Roger et al., 2017; Carvalheiro et al., 2019). Our results showed that bumble bees mainly visited the legumes, suggesting that they have specialized diets, which may explain why they are more susceptible to declines than flies and butterflies. For example, researchers have suggested that significant spatial changes in flowering plant species due to N enrichment would decrease the diversity of pollinators, such as bees and butterflies (Carvalheiro et al., 2019). Due to the mismatch in the scales at which pollinators and plants respond to the nutrient treatments, large-spatial-scale studies on the effects of different nutrient enrichments on the populations of pollinators will be investigated in the future.

### The impacts of nutrient additions on plant–pollinator interactions

Contrary to the results of previous studies, Burkle and Irwin (2009) found no effects of N addition on the identity and frequency of plant–pollinator interactions. Our results demonstrate that pollinators increasingly rely on fewer flowering plant species and that pollinators visit a reduced range of plant species. Thus, plant–pollinator interactions are sensitive to changes in flowering plant composition and flower abundance, and the variation in pollinator behavior among different plants that were affected by the N, P, and N + P enrichment treatments. These results support our third hypothesis that nutrient enrichment can change plant–pollinator interactions.

Plant–pollinator interaction networks always exhibit nested properties and are centered around a core of generalized plant and pollinator species (Bascompte et al., 2003; Jordano et al., 2006). Thus, changes in the composition of the core generalized plant species and floral abundance can directly affect the structure of the pollination network, such as nestedness (i.e., interactions between the most generalist plants and animals create a dense core that includes other specialized plants and animals in its community). The nested structure of the network can reduce the extent to which species and interactions are affected by perturbation (Bascompte et al., 2003; Pawar, 2014), indicating that pollination networks are highly tolerant of plant extinctions due to their nestedness structure.

Furthermore, the addition of N or P alone did not change the core generalized flowering plants, which may not change the nestedness of plant–pollinator interaction networks, because of the generalized characteristics of pollination networks (Waser et al., 1996; Johnson and Steiner, 2000) and the rapid turnover of interactions due to the loss and gain of non-core plant and pollinator species (Burkle and Irwin, 2009; CaraDonna et al., 2017). We also cannot rule out the possibility that the experimental plots were too small to attract different pollinators or that the pollinators are not specific for a given flowering plant species, as mentioned above. Although previous studies revealed that N and P enrichment reduces the biomass and abundance of legumes and the overall diversity of plant species in alpine grasslands (Ren et al., 2017; Luo et al., 2019), the loss of non-core species did not change the structure of the pollination networks (Weiner et al., 2011). Thus, changes in the composition and flower abundance of the core generalized flowering plant species due to the addition of N and P can influence the link distribution of the pollination networks.

## Conclusion

We tested the importance of nutrient enrichment for changes in pollination interactions by combining network theory and pollination ecology. Our study revealed that changes in flowering plant diversity and composition, both of which are reduced by nitrogen and phosphorus enrichment, alter plant–pollinator interactions in a Tibetan alpine grassland. Future experiments in manipulating nutritional resources, and the composition and abundance of plant and pollinator species on larger temporal and spatial scales will provide important insights into how nutrient enrichment affects the response of pollinator assemblages.

## Data availability statement

The original contributions presented in this study are included in the article/Supplementary Material, further inquiries can be directed to the corresponding authors.

## Author contributions

L-LW, Y-PY, Y-WD, and J-SH conceived the ideas and designed methodology. L-LW, FR, CZ, Z-HZ, and X-JH collected the data. L-LW analyzed the data. L-LW, Y-PY, and Y-WD led the writing of the manuscript. All authors contributed critically to the drafts and gave final approval for publication.
