# Supplementary material for: The effects of changes in flowering plant composition caused by nitrogen and phosphorus enrichment on plant–pollinator interactions in a Tibetan alpine grassland
Source: Front Plant Sci. 2022 Jul 25;13:964109. doi: 10.3389/fpls.2022.964109 (PMC9358526; doi:10.3389/fpls.2022.964109)
Supplement: Supplementary file 1 [file Data_Sheet_1.docx]

Supplementary Material

**The Effects of Changes in Flowering Plant Composition Caused by Nitrogen and Phosphorus Enrichment on Plant-Pollinator Interactions in a Tibetan Alpine Grassland**

Lin-Lin Wang, Fei Ren, Chan Zhang, Xiao-Juan Huang, Zhen-Hua Zhang, Jin-Sheng He, Yong-Ping Yang, and Yuan-Wen Duan

**The file includes:**

**Supplementary Table 1** List of plant species in our study. Nutrient enrichment: without nutrient fertilization (C), N enrichment (N), P enrichment (P), and N and P enrichment (N + P).

**Supplementary Table 2** The code of pollinator species. Nutrient enrichment: without nutrient fertilization (C), N enrichment (N), P enrichment (P), and N and P enrichment (N + P).

**Supplementary Table 3** Coefficient estimates from the final (reduced) piecewise structure equation model.

**Supplementary Table 4** Coefficient estimates from the full piecewise structure equation model.

**Supplementary Table 5** Key experimental studies demonstrating the effects of nutrient enrichment on pollinator communities.

**Supplementary Figure 1** Hypothesized direct effects of N enrichment and P enrichment, and the indirect effects mediated by plant and pollinator community (i.e., plant diversity, flower abundance, pollinator diversity and pollinator abundance), on vulnerability and generality. Paths a – d represent the direct effects of nutrient enrichment on vulnerability and generality, paths e – l represent the effects of nutrient enrichment on plant and pollinator community, paths m - o represent the effects of plant diversity on flower abundance, flower abundance on pollinator diversity, and pollinator diversity on pollinator abundance, respectively, while paths p – x represent the indirect effects of plant and pollinator community on vulnerability and generality.

**Supplementary Figure 2** The effects of nutrient enrichment on the richness and abundance of plants and pollinators. (A) the diversity of flowering plant species, (B) the number of flowers, (C) the diversity of pollinators, and (D) the number of pollinators. Differences between nutrient treatments was examined using a one-way analysis of variance, shown in each box. Lowercase letters on the bars indicate significant differences in nutrient supply at the 0.05 level.

**Supplementary Figure** **3** The effects of nutrient enrichment on the core generalized flowering plant species (A) and pollinator species (B) between the different nutrient treatments.

**Supplementary Figure 4** The full piecewise structural equation model results. The full model adequately fitted the data: χ^2^ = 1.629, df = 6, *P* = 0.95, AICc = -108.163. Vulnerability is the mean number of pollinator species per plant species. Generality is the mean number of plant species per pollinator species. Numbers on arrows are standardized path coefficients (scaled by their mean and standard deviation), and asterisks indicate statistical significance (***, *P* < 0.001; **, *P* < 0.01; *, *P* < 0.05). Red arrows, evidence for positive relationships; blue arrows, evidence for negative relationships; gray arrows, insufficient statistical evidence for path coefficients (*P* > 0.05). Width of the arrows shows the strength of the causal relationship, and *R*^2^ is the marginal *R*^2^, which indicates the variance explained by fixed effects in the mixed model.

**Supplementary Table 1** List of plant species in our study. Nutrient enrichment: without nutrient fertilization (C), N enrichment (N), P enrichment (P), and N and P enrichment (N + P).

| Fimily | Plant species | Control | N | P | NP |
| --- | --- | --- | --- | --- | --- |
| [Dipsacaceae](http://www.iplant.cn/info/Dipsacaceae?t=z) | *Morina kokonorica* | √ | √ | √ | √ |
| Fabaceae | *Oxytropis kansuensis* | √ |  | √ |  |
| Fabaceae | *Oxytropis ochrocephala* | √ |  | √ |  |
| Asteraceae | *Taraxacum maurocarpum* | √ | √ | √ | √ |
| Asteraceae | *Anaphalis lactea* | √ | √ | √ | √ |
| Asteraceae | *Aster farreri* | √ | √ | √ | √ |
| Asteraceae | *Saussurea subulisquama* | √ | √ |  |  |
| Gentianaceae | *Gentiana aristata* | √ | √ | √ |  |
| Gentianaceae | *Gentiana straminea* | √ | √ | √ | √ |
| Gentianaceae | *Gentianopsis paludosa* |  | √ |  |  |
| Ranunculaceae | *Ranunculus brotherusii* | √ | √ | √ | √ |
| Ranunculaceae | *Ranunculus membranaceus* | √ |  |  |  |
| Rubiaceae | *Galium verum* |  |  | √ |  |
| Rosaceae | *Potentilla saundersiana* | √ | √ | √ | √ |
| Rosaceae | *Potentilla fruticosa* | √ | √ | √ |  |
| Apiaceae | *Bupleurum smithii* |  |  |  | √ |
| Apiaceae | *Angelica nitida* | √ | √ | √ |  |
| Brassicaceae | *Descurainia sophia* | √ |  | √ | √ |
| Caryophyllaceae | *Stellaria media* |  |  |  | √ |
| Scrophulariaceae | *Euphrasia regelii* |  |  | √ |  |

**Supplementary Table 2** The code of pollinator species. Nutrient enrichment: without nutrient fertilization (C), N enrichment (N), P enrichment (P), and N and P enrichment (N + P).

| Codes | Order | Pollinator species | Control | N | P | NP |
| --- | --- | --- | --- | --- | --- | --- |
| C7 | Coleoptera | *Rhomborrhina splendida* | √ |  | √ |  |
| Ds64 | Diptera | *Archytas* sp1 |  |  | √ |  |
| DY67 | Diptera | *Bibio* sp2 | √ | √ | √ | √ |
| DY45 | Diptera | *Cheilosia* sp1 |  |  | √ |  |
| DC17 | Diptera | *Chrysomyia* sp2 |  |  | √ | √ |
| D7 | Diptera | *Conops* sp3 |  |  | √ |  |
| DY72 | Diptera | Diptera |  |  |  | √ |
| DY73 | Diptera | Diptera | √ | √ | √ | √ |
| DY76 | Diptera | Diptera |  |  |  | √ |
| DY77 | Diptera | Diptera | √ |  | √ | √ |
| DY54 | Diptera | *Empis* sp1 |  |  | √ | √ |
| DS29 | Diptera | *Eupeodes corollae* |  |  |  | √ |
| DY50 | Diptera | *Eurithia* sp1 | √ | √ | √ | √ |
| DY51 | Diptera | *Eurithia* sp2 | √ | √ | √ | √ |
| DC14 | Diptera | *Lucilia* sp1 | √ |  | √ |  |
| DC15 | Diptera | *Lucilia* sp2 | √ | √ | √ | √ |
| DY52 | Diptera | Muscidae sp1 | √ | √ | √ | √ |
| DY65 | Diptera | Muscidae sp2 | √ |  | √ | √ |
| DY47 | Diptera | Muscidae sp4 | √ |  |  | √ |
| DY49 | Diptera | Muscidae sp5 | √ | √ | √ | √ |
| DY41 | Diptera | Muscidae sp6 | √ |  |  |  |
| DY63 | Diptera | *Pales* sp1 | √ |  |  |  |
| DY56 | Diptera | *Platycheirus* sp2 |  | √ | √ |  |
| DY42 | Diptera | Sarcophagidae sp1 |  | √ |  |  |
| DY48 | Diptera | Sarcophagidae sp2 |  |  |  | √ |
| DY57 | Diptera | Sarcophagidae sp3 | √ |  |  | √ |
| DY38 | Diptera | Sarcophagidae sp5 |  |  |  | √ |
| DY61 | Diptera | *Sciara* sp1 | √ | √ | √ | √ |
| DS23 | Diptera | *Sphaerophoria viridaenea* |  |  |  | √ |
| DY46 | Diptera | *Sturmia* sp1 |  |  |  | √ |
| DS28 | Diptera | Syrphidae sp4 | √ |  | √ |  |
| D6 | Diptera | *Tachina fera* |  |  |  | √ |
| DY43 | Diptera | Tachinidae sp2 |  |  |  | √ |
| DY53 | Diptera | Tachinidae sp4 | √ | √ | √ | √ |
| DY59 | Diptera | Tachinidae sp5 |  |  | √ |  |
| DY68 | Diptera | Tachinidae sp7 |  |  | √ |  |
| B8 | Hemiptera | *holcostethus* sp |  |  | √ |  |
| H1 | Hymenoptera | *Apis mellifera* | √ | √ | √ |  |
| H22 | Hymenoptera | *Bombus supremus* | √ |  |  |  |
| B9 | Hymenoptera | Ichneumonidae sp2 |  |  | √ |  |
| B11 | Hymenoptera | Ichneumonidae sp3 | √ |  | √ | √ |
| B12 | Hymenoptera | Ichneumonidae sp4 | √ |  |  |  |
| D12 | Hymenoptera | *Tenthredo mesomela* | √ |  |  |  |
| LB18 | Lepidoptera | *Albulina orbitulus* | √ |  |  |  |
| LB17 | Lepidoptera | *Albulina* sp1 |  |  |  | √ |
| LB16 | Lepidoptera | *Anthocharis* sp1 |  |  | √ |  |
| LB15 | Lepidoptera | *Celastrina argiola* | √ | √ | √ | √ |
| LB20 | Lepidoptera | *Colias* sp2 | √ | √ | √ | √ |
| LB24 | Lepidoptera | *Cupido minimus* |  |  |  | √ |
| LB22 | Lepidoptera | *Everes argiades* |  |  | √ |  |
| LB19 | Lepidoptera | *Satyrium* sp3 |  |  |  | √ |
| LF1 | Lepidoptera | *Scythris* sp1 |  |  |  | √ |
| LB9 | Lepidoptera | *Speyeria aglaja* |  |  | √ |  |
| LB14 | Lepidoptera | *Zizina otis* |  | √ |  | √ |

**Supplementary Table 3** Coefficient estimates from the final (reduced) piecewise structure equation model.

| Response | Predictor | **Standard coefficient** | **Standard error** | ***P*** |
| --- | --- | --- | --- | --- |
| plantspecies | p | -0.147 | 0.5634 | 0.254 |
| plantspecies | n | -0.8087 | 0.5634 | 0 |
| plantflowers | plantspecies | -0.4791 | 1589.135 | 0.0678 |
| plantflowers | p | 0.4806 | 4237.694 | 0.0043 |
| plantflowers | n | 0.1 | 7126.542 | 0.6837 |
| insectspecies | p | 0.0107 | 1.5937 | 0.9589 |
| insectspecies | n | 0.2247 | 1.5937 | 0.2884 |
| insectnumbers | insectspecies | 0.6683 | 2.4904 | 0.0013 |
| insectnumbers | p | -0.1446 | 18.9007 | 0.3945 |
| insectnumbers | n | -0.2658 | 19.3956 | 0.1371 |
| vulnerability | insectnumbers | 0.5686 | 0.0043 | 0.0152 |
| vulnerability | insectspecies | 0.2283 | 0.0698 | 0.3237 |
| vulnerability | plantspecies | -0.163 | 0.1619 | 0.5964 |
| vulnerability | plantflowers | 0.417 | 0 | 0.1412 |
| vulnerability | n | -0.0849 | 0.6603 | 0.7582 |
| vulnerability | p | -0.0156 | 0.4889 | 0.9389 |
| generality | vulnerability | -0.6756 | 0.0834 | 0 |
| generality | plantflowers | -0.6543 | 0 | 0.0011 |
| generality | p | 0.1669 | 0.2521 | 0.2493 |
| generality | n | 0.3225 | 0.243 | 0.0303 |

**Supplementary Table 4** Coefficient estimates from the full piecewise structure equation model.

| Response | Predictor | Std.Estimate | Std.Error | P.Value |
| --- | --- | --- | --- | --- |
| plantspecies | p | -0.147 | 0.5634 | 0.254 |
| plantspecies | n | -0.8087 | 0.5634 | 0 |
| plantflowers | plantspecies | -0.4791 | 1589.1352 | 0.0678 |
| plantflowers | p | 0.4806 | 4237.6938 | 0.0043 |
| plantflowers | n | 0.1 | 7126.5423 | 0.6837 |
| insectspecies | plantflowers | -0.6299 | 0.0001 | 0.0659 |
| insectspecies | plantspecies | -0.2843 | 0.6663 | 0.4757 |
| insectspecies | p | 0.316 | 1.9243 | 0.2217 |
| insectspecies | n | 0.3018 | 2.6611 | 0.392 |
| insectnumbers | insectspecies | 0.6673 | 2.834 | 0.0042 |
| insectnumbers | plantflowers | -0.0047 | 0.0012 | 0.9879 |
| insectnumbers | plantspecies | 0.0196 | 8.5568 | 0.9547 |
| insectnumbers | p | -0.1391 | 26.5203 | 0.5581 |
| insectnumbers | n | -0.2474 | 35.2461 | 0.4355 |
| vulnerability | insectnumbers | 0.5686 | 0.0043 | 0.0152 |
| vulnerability | insectspecies | 0.2283 | 0.0698 | 0.3237 |
| vulnerability | plantspecies | -0.163 | 0.1619 | 0.5964 |
| vulnerability | plantflowers | 0.417 | 0 | 0.1412 |
| vulnerability | p | -0.0156 | 0.4889 | 0.9389 |
| vulnerability | n | -0.0849 | 0.6603 | 0.7582 |
| generality | vulnerability | -0.605 | 0.1305 | 0.0055 |
| generality | insectnumbers | -0.1875 | 0.0029 | 0.3334 |
| generality | insectspecies | 0.1213 | 0.0384 | 0.4772 |
| generality | plantspecies | 0.0254 | 0.0893 | 0.9114 |
| generality | plantflowers | -0.6583 | 0 | 0.0085 |
| generality | p | 0.1343 | 0.2761 | 0.396 |
| generality | n | 0.2793 | 0.3702 | 0.198 |

**Supplementary Table 5** Key experimental studies demonstrating the effects of nutrient enrichment on pollinator communities.

| Location | System | Methods | Key findings | References |
| --- | --- | --- | --- | --- |
| the Rocky Mountain Biological Laboratory, USA, 2900m, 38°57′N, 106°59′W | Meadows: Plants and pollinators | Three N treatments (2005-2007): control, low-N addition (1 g N m_2 year_1), or high-N addition (20 g N m_2 year_1); 24 plots (16 m2 each); one year of sampling. | no effects of nitrogen enrichment on the core group of generalist plants and pollinators or on plant-pollinator network. | Burkle, L., Irwin, R., 2009. The importance of interannual variation and bottom-up nitrogen enrichment for plant-pollinator networks. Oikos 118, 1816-1829. |
| the Ingleborough National Nature Reserve in the Yorkshire Dales, northern England, 300m, 54°12′N, 2°21′W. | Meadows: Plants, flower visitors, leaf miners and parasitoids | Four fertiliser treatments (1990-2016): mineral fertilizer (NPK; 25 kg/ha nitrogen, 12.5 kg/ha of P2O2 and K2O), farmyard manure (12 t/ ha, FYM), both fertilisers together, with a control; 72 plots (15m2 each); one year of sampling. | Insect species richness was unaffected by fertiliser treatment; fertilised plots had a significantly higher abundance of leaf miners and parasitoids and a significantly lower abundance of bumblebees.The plant–flower-visitor networks showed higher values of vulnerability with fertilizer addition | Villa-Galaviz, E., Smart, S.M., Clare, E.L., Ward, S.E., Memmott, J., 2021. Differential effects of fertilisers on pollination and parasitoid interaction networks. J. Anim. Ecol. 90, 404-414. |
| Netherlands  Country | Plants, bees and butterflies | historical dataset with information on species occurrences (plant, bees and butterflies) and nitrogen depo­sition over an 80 yr period (1930-2009). | the impacts of soil eutrophication on plant communi­ties propagate to higher trophic levels, but with a time-lag. the global increase in nitrogen availability plays an important role in the ongoing pollina­tor decline. | Carvalheiro, L.G., et al., 2019. Soil eutrophication shaped the composition of pollinator assemblages during the past century. Ecography 43, 209-221 |
| the Haibei Alpine Grassland Ecosystem Research Station, Qinghai, China.  3250m, 37°36′N, 101°12′E | Alpine Grassland  Plants and pollinators | Four nutrient addition treatments (2011-2019): control, N, P, N and P addition, 100 kg urea and 50 kg triple superphosphate per hectare per year; 24 plots (36 m2 each); one year of sampling | N and P co-addition can reshape flowering communities. This reshaping of flowering communities can promote changes in the interactions between plants and pollinators. | this study |


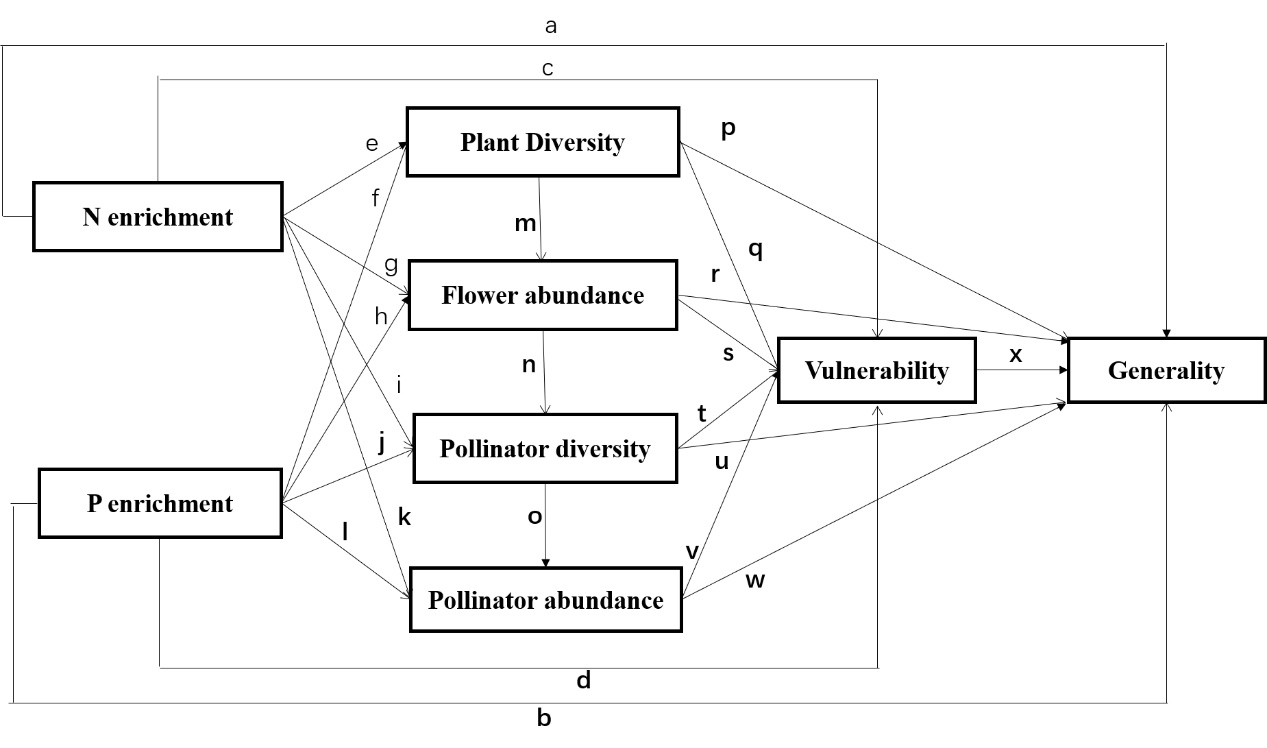


**Supplementary Figure** **1**. Hypothesized direct effects of N enrichment and P enrichment, and the indirect effects mediated by plant and pollinator community (i.e., plant diversity, flower abundance, pollinator diversity and pollinator abundance), on vulnerability and generality. Paths a – d represent the direct effects of nutrient enrichment on vulnerability and generality, paths e – l represent the effects of nutrient enrichment on plant and pollinator community, paths m - o represent the effects of plant diversity on flower abundance, flower abundance on pollinator diversity, and pollinator diversity on pollinator abundance, respectively, while paths p – x represent the indirect effects of plant and pollinator community on vulnerability and generality.

**
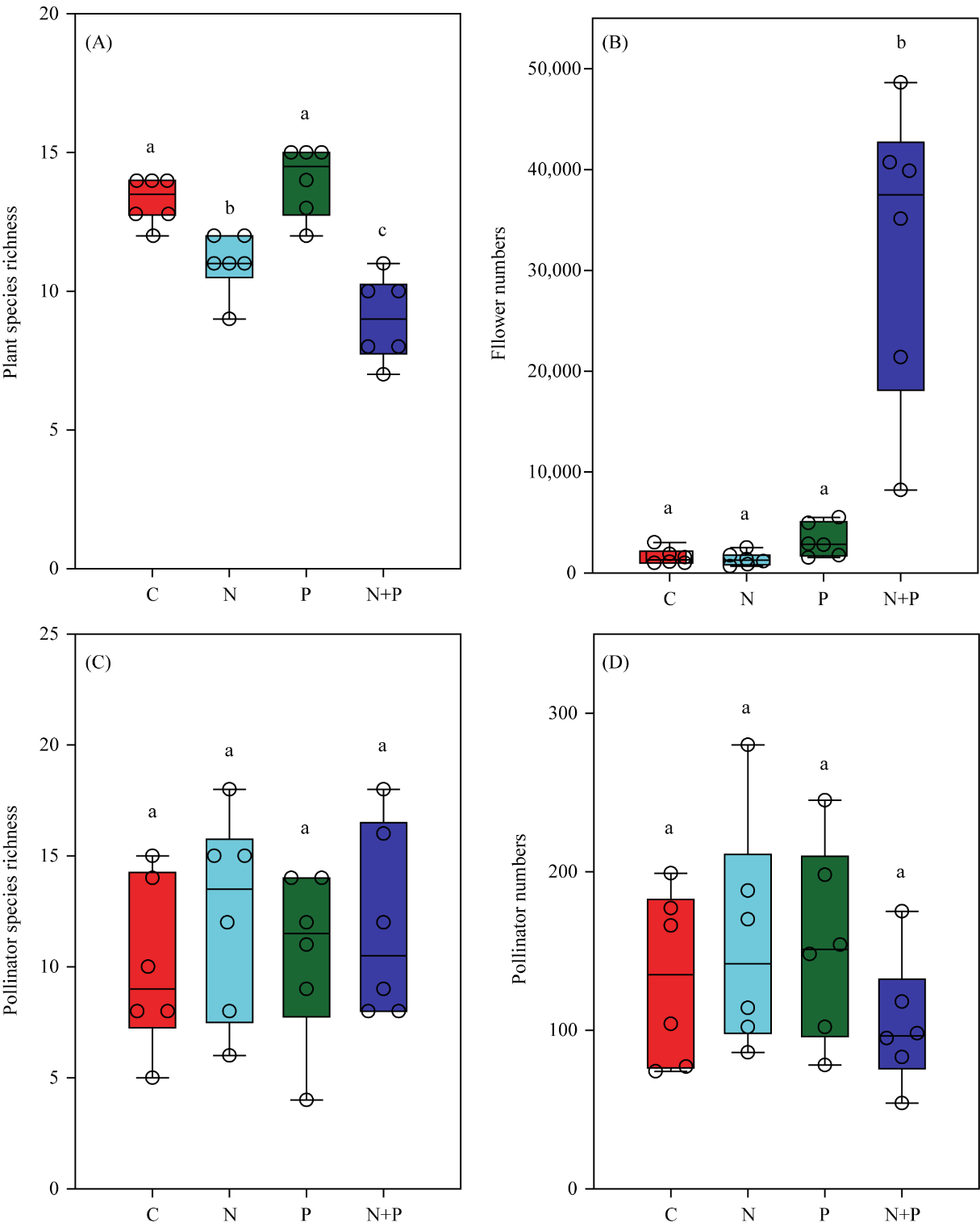
**

**Supplementary Figure 2** The effects of nutrient enrichment on the richness and abundance of plants and pollinators. (A) the diversity of flowering plant species, (B) the number of flowers, (C) the diversity of pollinators, and (D) the number of pollinators. Differences between nutrient treatments was examined using a one-way analysis of variance, shown in each box. Lowercase letters on the bars indicate significant differences in nutrient supply at the 0.05 level.


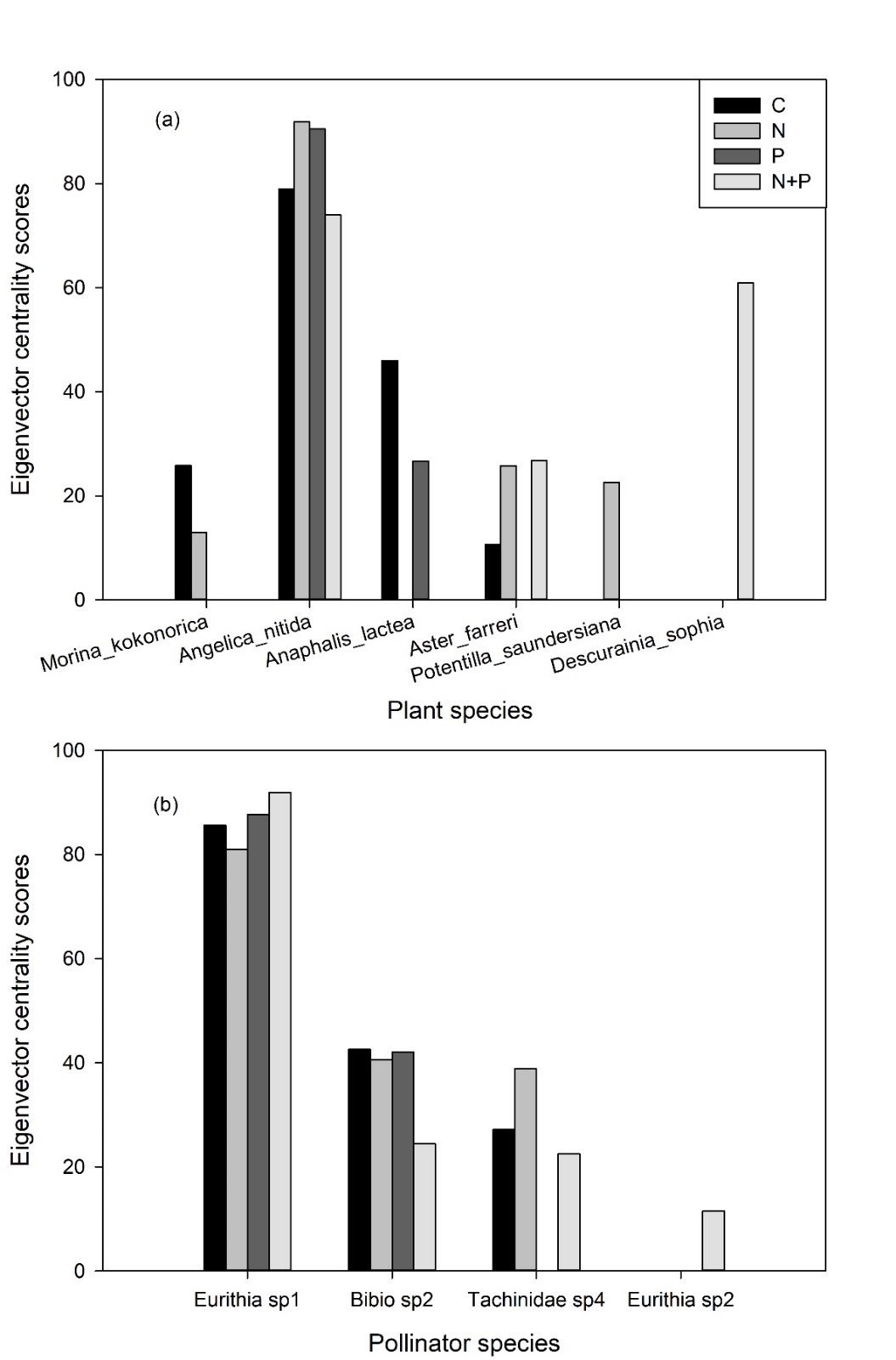


**Supplementary Figure 3** The effects of nutrient enrichment on the core generalized flowering plant species (A) and pollinator species (B) between the different nutrient treatments.


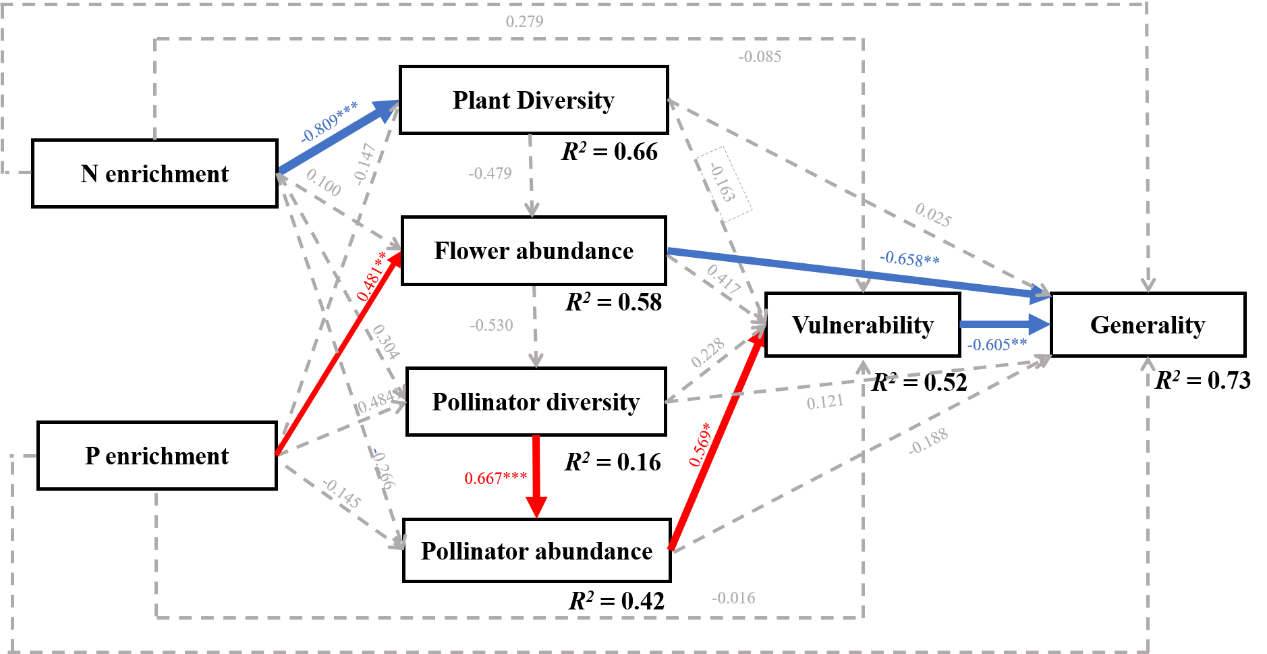


**Supplementary Figure 4**. The full piecewise structural equation model results. The full model adequately fitted the data: χ^2^ = 1.629, df = 6, *P* = 0.95, AICc = -108.163. Vulnerability is the mean number of pollinator species per plant species. Generality is the mean number of plant species per pollinator species. Numbers on arrows are standardized path coefficients (scaled by their mean and standard deviation), and asterisks indicate statistical significance (***, *P* < 0.001; **, *P* < 0.01; *, *P* < 0.05). Red arrows, evidence for positive relationships; blue arrows, evidence for negative relationships; gray arrows, insufficient statistical evidence for path coefficients (*P* > 0.05). Width of the arrows shows the strength of the causal relationship, and *R*^2^ is the marginal *R*^2^, which indicates the variance explained by fixed effects in the mixed model.
